# Broiler responses to copper levels and sources: growth, tissue mineral content, antioxidant status and mRNA expression of genes involved in lipid and protein metabolism

**DOI:** 10.1186/s12917-022-03286-5

**Published:** 2022-06-13

**Authors:** Helvio da Cruz Ferreira Júnior, Diego Ladeira da Silva, Bruno Reis de Carvalho, Haniel Cedraz de Oliveira, Jorge Cunha Lima Muniz, Warley Junior Alves, James Eugene Pettigrew, Simone Eliza Facione Guimarães, Gabriel da Silva Viana, Melissa Izabel Hannas

**Affiliations:** 1 Department of Animal Science, Universidade Federal de Viçosa, Viçosa, 36570900 Brazil; 2 Pettigrew Research Services, Arizona, 85646 USA; 3 Production Systems, Natural Resources Institute Finland (Luke), Jokioinen, Finland

**Keywords:** Gene expression, Growth, Tissue mineralization, Trace minerals

## Abstract

**Background:**

Five hundred 8-d old male broilers Cobb500 were randomly allotted into 10 treatments in factorial arrangement with 5 Cu levels (0, 4, 8, 12, and 16 mg/kg), and 2 sources (Cu proteinate, CuPro and Cu sulphate, CuSO4.5H2O) for a 10-d-experiment.

**Results:**

Feed conversion ratio (FCR) was better (*P* < 0.05) in CuPro fed chicks compared with CuSO4.5H2O group. Average daily feed intake (ADFI) decreased linearly (*P* < 0.05) as dietary Cu increased. A quadratic response (*P* < 0.05) to Cu levels was found for FCR, being optimized at 9.87 and 8.84 mg Cu/kg in CuPro and CuSO4.5H2O diets, respectively. Copper supplementation linearly increased liver Cu content (*P* < 0.05) and tended to linearly increase (*P* = 0.07) phosphorus (*P*) and copper in tibia. Manganese and zinc were higher (*P* < 0.05) in tibia of CuPro fed birds. Broilers fed CuPro exhibited lower liver iron (*P* < 0.05) content, lower activities of Cu, Zn superoxide dismutase (CuZnSOD) in breast muscle and liver, and glutathione peroxidase in liver. Glutathione peroxidase reduced linearly (*P* < 0.05) with CuPro levels and increased linearly (*P* < 0.05) with CuSO4.5H2O levels and were lower (*P* < 0.05) in all CuPro levels in breast muscle. Breast muscle malondialdehyde concentration tended to be higher (*P* = 0.08) in broilers fed CuSO4.5H2O. Copper levels linearly increased (*P* < 0.05) metallothionein (MT) and malate dehydrogenase (MDH) expression in liver, and six-transmembrane epithelial antigen of the prostate-1 (STEAP-1) in the intestine. Copper elicited a quadratic response (*P* < 0.050) in AKT-1 and mammalian target of rapamycin (mTOR) in breast muscle, CuZnSOD in liver and antioxidant 1 copper chaperone (ATOX 1) in intestine. Broilers fed CuPro exhibited higher mRNA expression of mTOR in muscle breast and lower CuZnSOD in liver and ATOX 1 in intestine. Interaction (*P* < 0.05) between levels and sources was found in mRNA expression for GSK-3β, MT, and CuZnSOD in breast muscle, FAS and LPL in liver and MT and CTR1 in intestine.

**Conclusions:**

CuPro showed beneficial effects on feed conversion and bone mineralization. Organic and inorganic Cu requirements are 9.87 and 8.84 mg Cu/kg, respectively.

**Supplementary Information:**

The online version contains supplementary material available at 10.1186/s12917-022-03286-5.

## Background

Copper (Cu) is an indispensable trace element for poultry maintenance, growth, health, and survival. As co-factor for several metalloenzymes, Cu is involved in a plethora of biological processes, which include mitochondrial respiration, erythropoiesis, connective tissue maturation, free radical scavenging, hormone secretion pathways and immune system defenses, among others [1–5]. Deficiency of Cu compromises performance objectives and immune functions [6–8] and leads to skeletal system disorders in growing broiler chickens [9, 10].

In the last revised edition of Nutrient Requirements of Poultry [11], the Poultry Subcommittee of the National Research Council estimated the broiler requirements for Cu as 8 mg/kg diet to prevent deficiency symptoms and support growth. Despite its importance as a reference, this estimate remains a matter of criticism because critical source data were published almost four decades ago [12] when the birds and their productive performance were far different from those currently used.

Supplemental Cu has been conventionally provided as either copper sulfate pentahydrate (CuSO_4_.5H_2_O) or, to a lesser extent, other inorganic salts such as oxides and carbonate. These inorganic sources offer low cost, but they are less bioavailable than some organic sources [13–15], which is generally compensated by increasing the safety margin of supplementation to avoid nutritional deficiencies [16]. Providing excessive Cu in diets may compromise the utilization of other minerals such as calcium, phosphorous and zinc by forming insoluble complexes with binding agents (e.g., phytate) or competing for uptake sites in intestinal mucosa [17–20]. Once unavailable for intestinal absorption, such complexes are inevitably excreted and may increase environmental pollution of soils and water resources, which could damage biodiversity and ecosystems. Excessive dietary Cu has been reported to increase oxidative stress in broiler cells and tissues by increasing the production of reactive oxygen species leading to DNA fragmentation, peptide bond breakage and programmed cell death [21, 22].

Organic trace minerals have been extensively argued to be more efficiently absorbed than inorganic salts, and therefore, less excreted into environment [18, 23]. Due to their chemical structure, organic trace minerals avoid the formation of insoluble complexes with binding agents like phytate, other minerals, and dietary constituents in the gastrointestinal tract [24–26]. Copper chelates have been shown to increase the digestibility of nutrients and boost immune responses in broilers [27, 28]. There is evidence that dietary inorganic Cu can influence the expression of genes related to absorption and lipid and protein metabolism [1, 29, 30]. Organic Cu may provide the same effect of altering gene expression linked to lipid metabolism, protein and absorption and Cu linked genes [30, 31].

Generally, in feeding assays conducted to investigate broiler responses to organic Cu, all the other trace minerals such as zinc (Zn), manganese (Mn), iron (Fe), and selenium (Se) have been provided as inorganic salts. We have two main concerns about this practice. Firstly, commercial flocks are typically fed diets containing trace minerals supplied entirely as inorganic salts or as organic chelates, so responses to dietary levels of those minerals are most appropriately measured in the typical environment. Secondly, considering the interactions among trace minerals regarding intestinal absorption and metabolic pathways, physiological responses and gene expression could be also affected by the source of the trace minerals and supplemental copper levels. We hypothesized that trace mineral source and copper levels may provide different responses in animal growth, mineral concentration, antioxidant status and gene expression depending on the source of the other trace minerals added to the diet. Due to Cu antimicrobial effects, researchers have put efforts towards investigating dietary Cu supplementation at levels that exceed requirements [28, 32, 33], including pharmacological levels [34, 35]. Nonetheless, not so much has been done to determine broiler requirements for inorganic sources and organic Cu in feeds supplemented with inorganic and organic trace minerals, respectively. To the best of our knowledge, this is the first research in which such approach is considered, and the novel findings obtained can potentially contribute to elucidate which would be the best strategy to optimize trace minerals in practical broiler feeding programs.

The objectives of this research were: 1) to assess the effects of different dietary supplemental Cu levels as organic or inorganic in the same source of trace mineral supplement on growth, tissue mineralization, antioxidant status and gene expression linked with absorption, lipid, and protein metabolism, and 2) to estimate the optimal supplemental organic and inorganic Cu levels for chick performance.

## Results

The analyzed concentrations of Cu in experimental diets were 1.30, 6.09, 9.23, 13.3 and 17.3 mg/kg for diets supplemented with CuSO_4_.5H_2_O, and 1.18, 3.42, 8.97, 11.9 and 15.5 mg/kg for diets containing CuPro as the Cu source. These values were near the expected content of 0, 4, 8, 12, and 16 mg/kg (Table 1).

**Table 1:** Analyzed concentration of copper and trace minerals in experimental diets (as fed basis)

Mineral sources^a^	Trace minerals^b^	Copper supplementation in basal diet^c^ (mg/kg)				
		0	4	8	12	16
**Organic**	Cu	1.18	3.42	8.97	11.9	15.5
	Fe	112	116	112	111	111
	Mn	63.0	64.0	61.0	64.0	65.0
	Zn	49.0	48.0	50.0	48.0	48.0
**Inorganic**	Cu	1.30	6.09	9.23	13.3	17.3
	Fe	93.0	101	122	124	110
	Mn	57.0	62.0	62.0	66.0	58.0
	Zn	44.0	43.0	39.0	40.0	49.0

^a^Organic: copper proteinate (CuPro) and organic trace minerals; Inorganic: copper sulphate (CuSO_4._5H_2_O) and inorganic trace minerals

^b^Value determined by analysis. Each value based on 10 replicates

^c^Copper supplementation levels were obtained by adding 0; 4; 8; 12; and 16 mg Cu/kg diet to a semi-purified diet containing 1.32 mg Cu/kg

### Performance

No interaction effects (*P* > 0.05) between the supplemental levels and sources investigated on broiler performance responses were found (Table 2). Broiler FCR was lower (*P* < 0.05) in chicks fed CuPro and organic trace minerals than in those fed inorganic trace minerals. There was a tendency (*P* = 0.053) for greater ADFI in broilers fed CuSO_4_.5H_2_O and inorganic than organic trace minerals. Graded supplemental Cu levels elicited a linear increase in (*P* < 0.05) ADFI and a quadratic response (*P* < 0.05) in BW, ADG, and FCR. According to the polynomial quadratic regression model, the supplemental dietary Cu levels which optimized broiler responses considering both sources combined and each one separately, where the quadratic term significant, is presented in Table 3. In CuPro supplemented diets, the supplemental level of Cu, which optimized BW and ADG was estimated at 7.44 and 7.47 mg of Cu/kg diet, respectively (Table 3). Feed conversion was optimized at similar levels, 9.87 and 8.84 mg of Cu/kg in broilers fed CuPro and CuSO_4_.5H_2_O supplemented diets, respectively.

**Table 2:** Growth performance of 17 d old broilers chicks fed different copper levels and sources

Item	Copper levels (mg/kg)					Source		SEM^a^	Source	Level	Source × Level	L^b^	Q^c^
	0	4	8	12	16	Inorganic	Organic						
BW, g/bird	505	512	510	505	503	505	509	1.319	0.164	0.169	0.921	0.282	0.044
ADG, g/bird/day	33.7	34.4	34.3	33.8	33.5	33.7	34.1	0.132	0.159	0.172	0.923	0.283	0.045
ADFI, g/bird/day	44.4a	44.2ab	43.8 ab	43.1b	43.5ab	44.1	43.5	0.158	0.053	0.041	0.813	< 0.01	0.531
FCR, g/g	1.31a	1.28ab	1.27b	1.27b	1.30ab	1.31	1.27	< 0.01	< 0.01	0.013	0.953	0.077	< 0.01

Abbreviations: *BW* Body weight, *ADG* Average daily gain, *ADFI* Average daily feed intake, *FCR* Feed conversion ratio

^a^Standard error of means

^b^Linear effect of dietary copper levels

^c^Quadratic effect of dietary copper levels

Organic: copper proteinate (CuPro) and organic trace minerals; Inorganic: copper sulphate (CuSO_4._5H_2_O) and inorganic trace minerals

^a^^−^^b^Means with a different superscript in a row are different (*P* < 0.05)

**Table 3:** Optimum supplemental copper (Cu) level for broiler chicks considering sources individually or combined

Item	Regression Eqs. ^1^	Optimal supplemental Cu level. mg/kg^2^	*P*-value	Coefficient of determination (R^2^)
***Body weight***				
Copper levels	y = − 0.1003x^2^ + 1.3533x + 505.73	6.75	0.042	5.36
CuPro	y = − 0.1296x^2^ + 1.9294x + 505.78	7.44	0.066	6.73
***Average daily gain***				
Copper levels	y = − 0.0101x^2^ + 0.1352x + 33.81	6.76	0.045	5.33
CuPro	y = − 0.0129x^2^ + 0.1926x + 33.82	7.47	0.067	6.63
***Feed conversion ratio***				
Copper levels	y = 0.00049x^2^ − 0.0091x + 1.3182	9.31	0.002	11.4
CuPro	y = 0.00044x^2^ − 0.0088x + 1.3038	9.87	0.041	13.7
CuSO_4._5H_2_O	y = 0.00054x^2^ − 0.0095x + 1.3325	8.84	0.014	13.3

CuPro (organic), copper proteinate and organic trace minerals; CuSO_4_.5H_2_O (inorganic), copper sulphate and inorganic trace minerals

^1^Regression equations obtained from fitting performance data to polynomial quadratic regression model Y is dependent variable and X is the supplementation Cu concentration in the corresponding diet (mg/kg)

^2^Cu/kg diet to a semi-purified diet containing 1.32 mg Cu/kg

### Tissue mineral concentrations

Overall, neither trace mineral sources nor dietary Cu supplementation, nor their interaction, affected (*P* > 0.05) mineral content in breast muscle (Table 4). No differences in the liver Cu concentration were detected (*P* > 0.05) between broilers fed diets supplemented with inorganic versus organic trace minerals. Increasing levels of dietary Cu supplementation linearly increased (*P* < 0.05) Cu concentration in liver. Broilers fed CuPro and organic trace minerals had lower concentrations of Fe (*P* < 0.05) in liver compared with birds fed inorganic trace minerals. As dietary Cu supplementation increased, there was a tendency for a linear increase (*P* < 0.10) in the concentration of Cu and P and a tendency for a quadratic increase (*P* < 0.10) in the concentration of Mn in broiler tibia. The concentrations of Mn and Zn in the tibia of broilers fed CuPro and organic trace minerals were greater (*P* < 0.05) compared with broilers fed CuSO_4_.5H_2_O and inorganic trace minerals.

**Table 4:** Mineral concentration (dry matter) on tissues of 17 d old broiler chicks fed different copper levels and sources

Item	Copper levels, mg/kg					Source		SEM^1^	Source	Level	Source × Level	L^2^	Q^3^
	0	4	8	12	16	Inorganic	Organic						
***Breast muscle***													
Calcium, g/kg	91.5	97.7	87.4	89.2	94.5	90.7	93.4	2.09	0.524	0.540	0.426	0.877	0.569
Phosphorus, g/kg	9.60	9.52	9.63	9.33	9.54	9.56	9.48	64.7	0.475	0.621	0.673	0.502	0.767
Copper, mg/kg	1.72	1.70	1.62	1.60	1.74	1.69	1.65	0.031	0.524	0.508	0.405	0.782	0.144
Zinc, mg/kg	19.0	18.9	18.6	18.6	18.7	18.8	18.7	0.132	0.863	0.798	0.629	0.314	0.545
Iron, mg/kg	17.5	17.7	16.6	17.1	18.9	17.7	17.5	0.307	0.736	0.164	0.554	0.271	0.067
Manganese, mg/kg	0.575	0.593	0.714	0.620	0.650	0.624	0.637	0.022	0.762	0.315	0.960	0.272	0.275
***Liver***													
Calcium, g/kg	126	125	142	115	123	122	130	3.17	0.175	0.100	0.726	0.495	0.297
Phosphorus, g/kg	9.79	9.78	9.93	9.46	9.73	9.87	0.960	0.111	0.249	0.757	0.685	0.577	0.954
Copper, mg/kg	8.02^b^	8.67^ab^	8.71^ab^	8.36^ab^	9.15^a^	8.54	8.62	0.129	0.766	0.077	0.692	0.033	0.907
Zinc, mg/kg	54.3	54.1	52.7	51.9	54.3	53.5	53.4	0.683	0.915	0.749	0.412	0.667	0.326
Iron, mg/kg	266	267	249	253	261	287^b^	231^a^	7.88	< 0.01	0.930	0.470	0.663	0.584
Manganese, mg/kg	8.46	8.01	7.96	7.94	8.23	7.96	8.28	0.129	0.233	0.648	0.247	0.564	0.168
***Tibia***													
Calcium, g/kg	144	145	147	146	148	148	144	1.30	0.199	0.847	0.834	0.261	0.949
Phosphorus, g/kg	69.8	71.4	70.2	70.8	73.1	71.6	70.5	0.476	0.266	0.212	0.305	0.074	0.398
Copper, mg/kg	3.90	4.04	4.02	4.03	4.09	4.01	4.02	0.027	0.806	0.300	0.621	0.064	0.594
Zinc, mg/kg	186	184	186	185	184	181^b^	189^a^	1.61	< 0.01	0.989	0.107	0.766	0.895
Iron, mg/kg	197	197	195	192	201	195	197	2.87	0.780	0.897	0.474	0.909	0.488
Manganese, mg/kg	6.42	6.13	6.07	6.09	6.30	6.02^b^	6.38^a^	0.073	0.014	0.467	0.719	0.554	0.075

^1^Standard error of means

^2^Linear effect of dietary copper levels

^3^Quadratic effect of dietary copper levels

Organic: copper proteinate (CuPro) and organic trace minerals; Inorganic: copper sulphate (CuSO_4._5H_2_O) and inorganic trace minerals

^a^^−^^b^Means with a different superscript in a row are different (*P* < 0.05)

### Antioxidant enzyme activities and lipid oxidation

Chicks fed CuPro and organic trace minerals exhibited a lower activity (*P* < 0.05) of SOD in breast muscle and liver and GSH-Px in liver compared with birds fed diets containing CuSO_4_.5H_2_O and inorganic trace minerals (Table 5). An interaction between trace mineral sources and Cu levels on GSH-Px activity in chick breast muscle was found (*P* < 0.05). As CuPro supplementation increased in diets with organic trace minerals, breast muscle GSH-Px activity decreased linearly (*P* < 0.05), whereas a linear increase occurred in chicks fed CuSO_4_.5H_2_O and inorganic trace minerals diets (*P* < 0.05). Regardless of the level of Cu supplementation, GSH-Px activity in breast muscle of chicks fed CuSO_4_.5H_2_O and inorganic trace minerals was higher (*P* < 0.01) than in birds fed CuPro and organic trace minerals. Malondialdehyde concentration in breast muscle tended to be slightly lower (*P* = 0.08) in chicks fed diets with CuPro and organic trace minerals.

**Table 5:** Antioxidant enzyme activity and malondialdehyde concentration in 17 d old broiler chicks fed different copper levels and sources

Item	Copper levels, mg/kg					Mean		SEM^1^	Source	Level	Source × Level	L^2^	Q^3^
	0	4	8	12	16	Inorganic	Organic						
***Breast muscle***													
Superoxide dismutase, g/g pro	1.62	1.61	1.66	1.66	1.62^b^	1.66^a^	1.61^b^	0.013	0.050	0.471	0.173	0.499	0.266
Glutathione Peroxidase, IU/g pro	398	391	358	403	404	491^a^	291^b^	14.6	< 0.01	0.303	0.001	0.653	0.143
* Inorganic*	*436*	*507*	*454*	*515*	*541*			11.9				< 0.01	0.795
* Organic*	*361*	*274*	*263*	*291*	*268*			12.2				0.028	0.068
* p-value*^4^	< *0.01*	< *0.01*	< *0.01*	< *0.01*	< *0.01*								
MDA, mg/kg	0.0100	0.0161	0.0161	0.0161	0.0162	0.0181	0.0117	0.002	0.083	0.738	0.385		
***Liver***													
Superoxide dismutase, g/g pro	363	327	292	313	320	341^a^	306^b^	8.67	0.033	0.101	0.164	0.081	0.042
Glutathione Peroxidase, IU/g pro	2624	2608	2592	2475	2510	2951^a^	2173^b^	51.1	< 0.01	0.170	0.193	0.030	0.991

^1^Standard error of means

^2^Linear effect of dietary copper levels

^3^Quadratic effect of dietary copper levels

^4^Effect of the source (organic and inorganic) on Glutathione Peroxidase activity by dietary copper levels

Organic: copper proteinate (CuPro) and organic trace minerals; Inorganic: copper sulphate (CuSO_4._5H_2_O) and inorganic trace minerals; MDA: malondialdehyde

^a^^−^^b^Means with a different superscript in a row are different (*P* < 0.05)

### mRNA expression in the breast muscle, liver, and intestine

The *P-*values from analyses of variance of mRNA expression of genes in breast muscle, liver and jejunum of chicks fed supplemental Cu levels and different sources of trace minerals are detailed in Table 6.

**Table 6:** mRNA expression in tissues of 17 d old broilers fed different copper levels and sources

	Breast Muscle			Liver			Intestine (jejunum)		
Genes	Source	Level	S vs L	Source	Level	S x L	Source	Level	S vs L
AKT 1	0.1169	0.0450	0.1569	––	––	––	––	––	––
mTOR	0.0046	< 0.001	0.1561	––	––	––	––	––	––
GSK 3B	0.0871	0.0057	0.0499	––	––	––	––	––	––
MT	0.8910	0.2924	0.0124	0.5524	0.0036	0.3867	0.2718	< 0.001	< 0.001
CuZnSOD	0.0189	< 0.01	0.0028	< 0.001	< 0.001	0.2038	––	––	––
FAS	––	––	––	0.0058	0.0446	0.0097	––	––	––
LPL	––	––	––	0.1020	0.0002	0.0002	––	––	––
MDH	––	––	––	0.7833	0.0170	0.0535	––	––	––
CTR1	––	––	––	––	––	––	0.0530	0.0010	0.0144
ATOX1	––	––	––	––	––	––	0.0003	< 0.001	0.1027
STEAP1	––	––	––	––	––	––	0.2745	0.0002	0.3132

Abbreviations: *AKT 1* Protein kinase serine-threonine 1, *mTOR* Mechanistic target of rapamycin, *GSK 3B* Glycogen synthase kinase, *MT* Metallothionein, *CuZnSOD* Superoxide dismutase copper-zinc, *FAS* Fatty Acid Synthetase, *LPL* Lipoprotein lipase, *MDH* Malate dehydrogenase, *CTR1* Copper transporter 1, *ATOX1* Antioxidant target 1 copper chaperone, *STEAP1* Transmembrane Epithelial Antigene of the Prostate, *S* Source, *L* Level

AKT-1, mTOR, GSK-3β, MT, and CuZnSOD mRNA expression in the breast muscle

**Fig. 1:**
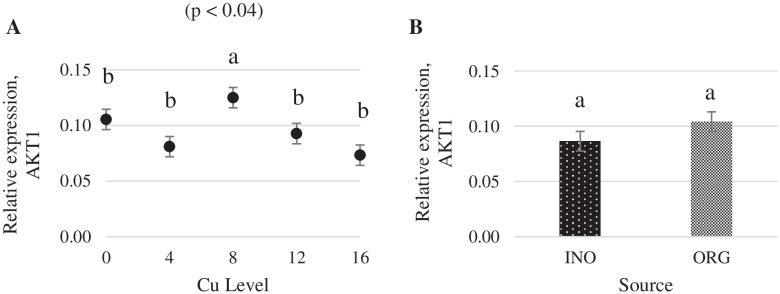
Copper levels and sources on mRNA expression of AKT-1 gene in broiler breast muscle. By the pooled means of the factors (Cu Level and Source): (**A**) Cu levels in mg/kg of the grouping of sources (INO and ORG), (**B**) Sources of grouping of Cu levels. Mean values without equal letters (**a-b**) differ between groups of Cu level (*P* < 0.05). AKT-1, serine-threonine 1 protein kinase; INO, source of sinorganic trace minerals; ORG, source of organic trace minerals

**Fig. 2:**
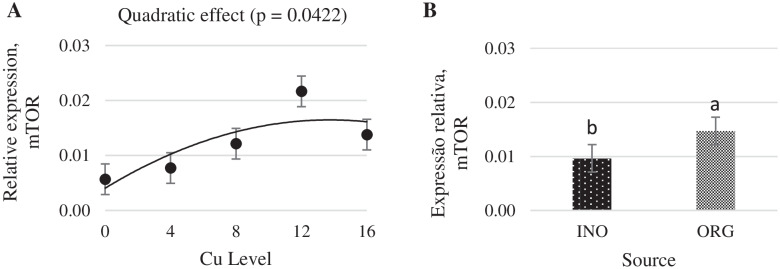
Copper levels and sources on mRNA expression of mTOR gene in broiler breast muscle. By the pooled means of the factors (Cu Level and Source): (**A**) Cu levels in mg/kg of the grouping of sources (INO and ORG), (**B**) Sources of grouping of Cu levels. Mean values without equal letters (**a-b**) differ between source groups (*P* < 0.05). mTOR, mechanistic target of rapamycin; INO, source of inorganic trace minerals; ORG, source of organic trace minerals

**Fig. 3:**
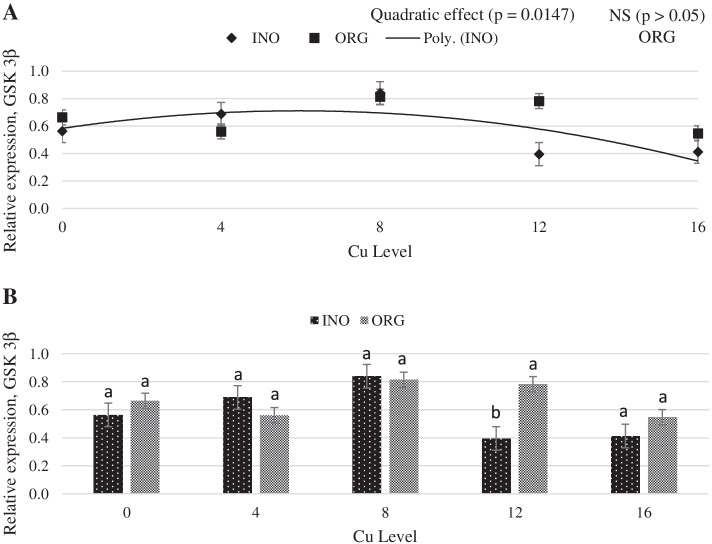
Copper levels and sources on mRNA expression of GSK-3β gene in broiler breast muscle. By the unfolding of the source vs copper level interaction: (**A**) Cu levels in mg/kg diet within the INO and ORG sources, (**B**) Sources within each Cu level. Mean values without equal letters (**a-b**) differ between groups (*P* < 0.05). GSK-3β, glycogen synthase kinase 3β; INO, inorganic trace minerals; ORG, organic trace minerals

**Fig. 4:**
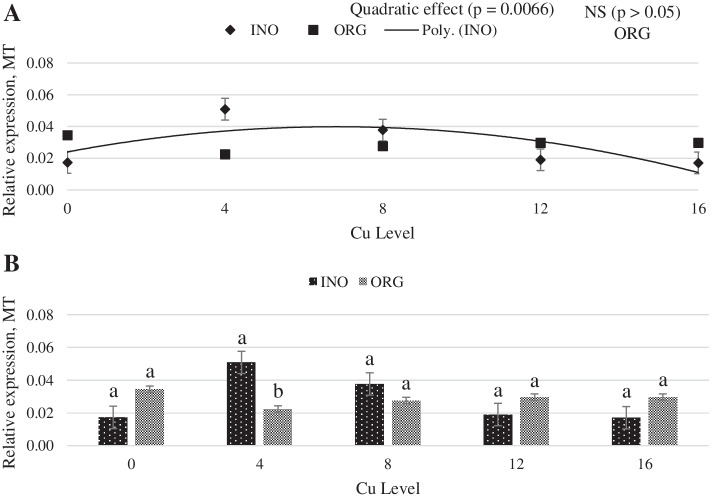
Copper levels and source on mRNA expression of MT gene in broilers breast muscle. By the unfolding of the source vs copper level interaction: (**A**) Cu levels in mg/kg within the INO and ORG source, (**B**) Sources within each copper level in mg/kg. Mean values without equal letters (**a-b**) differ between groups (*P* < 0.05). *MT* Metallothionein, *INO* Source of inorganic trace minerals, *ORG* Source of organic trace minerals

**Fig. 5:**
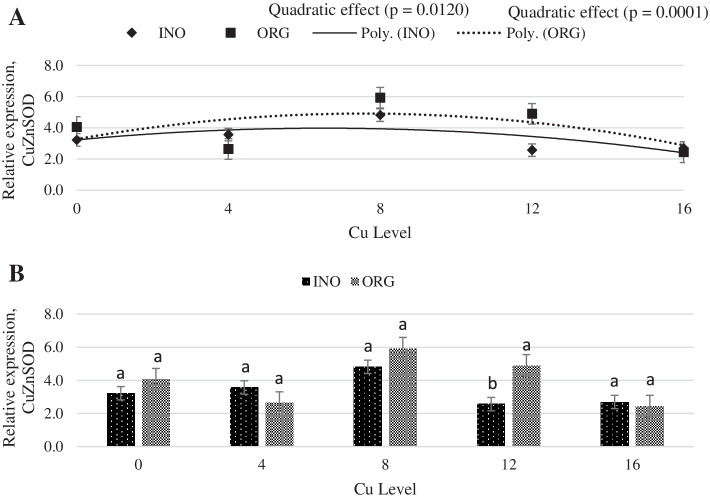
Copper levels and sources on mRNA expression of CuZnSOD gene in broiler breast muscle. By the unfolding of the source vs copper level interaction: (**A**) Cu levels in mg/kg within the INO and ORG source, (**B**) Sources within each copper level in mg/kg. Mean values without equal letters (**a-b**) differ between groups (*P* < 0.05). CuZnSOD, copper-zinc superoxide dismutase; INO, source of inorganic trace minerals; ORG, source of organic trace minerals

As shown in Fig. 1, regardless of the source investigated, the mRNA expression of protein kinase serine-threonine 1 (AKT-1) in chick breast muscle was higher at 8 mg Cu/kg diet (*P* < 0.05) than at other levels. Dietary Cu supplementation level elicited a quadratic response (P < 0.05) on the mRNA expression of mechanistic target of rapamycin (mTOR) in chick breast muscle, and chicks fed CuPro and organic trace minerals exhibited a higher (*P* < 0.05) mTOR expression compared with inorganic trace minerals fed chicks (Fig. 2). Interactive effects between supplemental Cu levels and trace minerals sources were found for the mRNA expression of glycogen synthase kinase (GSK-3β), metallothionein (MT), and copper-zinc superoxide dismutase (CuZnSOD) (*P* < 0.05). There was a quadratic effect (*P* < 0.05) of the supplemental Cu levels on the mRNA expression of GSK-3β (*P* < 0.05) when Cu was provided as CuSO_4_.5H_2_O with inorganic trace minerals, whereas no Cu-level effects (*P* > 0.05) were found in chicks fed CuPro with organic trace minerals diets (Fig. 3). At 12 mg Cu/kg diet, GSK-3β mRNA expression was greater in chicks fed CuPro and organic trace minerals diets than those fed CuSO_4_.5H_2_O and inorganic trace minerals (Fig. 3). Likewise, MT mRNA expression in chick breast muscle was quadratically influenced by level of CuSO_4_.5H_2_O supplementation (*P* < 0.05) but no effects of Cu levels (*P* > 0.05) were detected in chicks fed CuPro (Fig. 4). Chicks fed the diet supplemented with 4 mg Cu/kg as CuSO_4_.5H_2_O with inorganic trace minerals exhibited higher (*P* < 0.05) MT mRNA expression compared with CuPro and organic trace minerals (Fig. 4). A quadratic effect (*P* < 0.05) of the supplemental Cu levels on mRNA expression of CuZnSOD occurred in chicks fed either CuPro or CuSO_4_.5H_2_O (Fig. 5). The mRNA expression of CuZnSOD was higher (*P* < 0.05) in chicks fed diets supplemented with CuPro at 12 mg Cu/kg and organic trace minerals compared with chicks fed the same level provided as CuSO_4_.5H_2_O and inorganic trace minerals (Fig. 5).

### MT, CuZnSOD, FAS, LPL and MDH mRNA expression in the liver

**Fig. 6:**
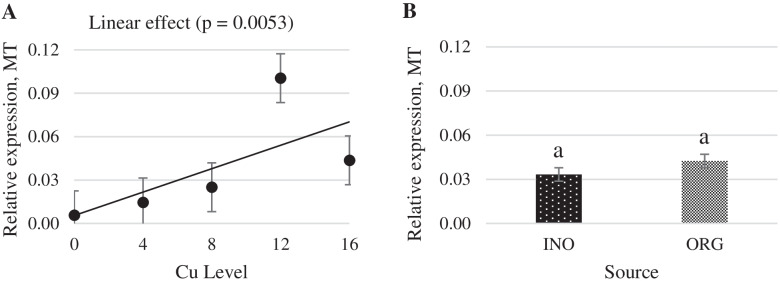
Copper levels and sources on mRNA expression of MT gene in the broiler liver. By the pooled means of the factors (Cu Level and Sources): (**A**) Copper levels in mg/kg of the grouping of sources (INO and ORG), (**B**) Sources of grouping of Cu levels. Mean values without equal letters (**a-b**) differ between groups (*P* < 0.05). *MT* Metallothionein, *INO* Source of inorganic trace minerals, *ORG* Source of organic trace minerals

**Fig. 7:**
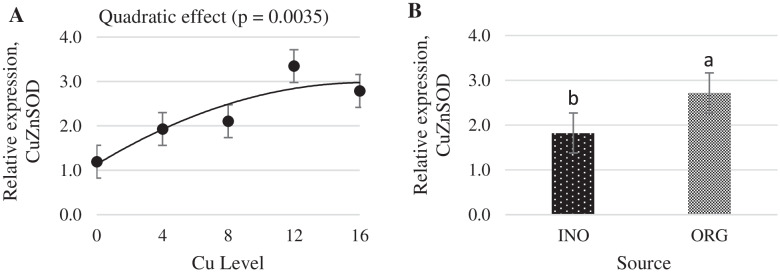
Copper levels and sources on mRNA expression of CuZnSOD gene in the broiler liver. By the pooled means of the factors (Cu Level and Sources): (**A**) Cu levels in mg/kg of the grouping of sources (INO and ORG), (**B**) Source of grouping of Cu levels. Mean values without equal letters (**a-b**) differ between groups (*P* < 0.05). *CuZnSOD* Copper-zinc superoxide dismutase, *INO* Source of inorganic trace minerals, *ORG* Source of organic trace minerals

**Fig. 8:**
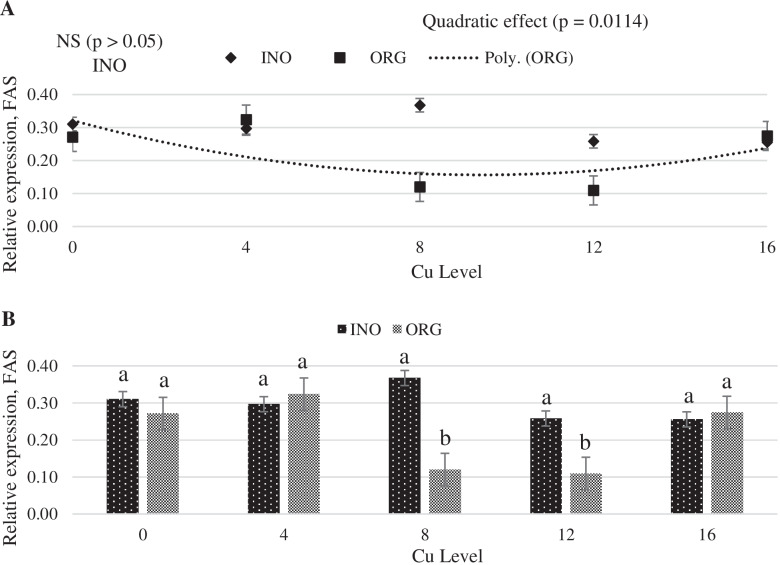
Copper levels and sources on mRNA expression of FAS gene in the broiler liver. By the unfolding of the interaction Cu levels vs sources. (**A**) Cu levels in mg/kg within the INO and ORG sources, (**B**) Sources within each Cu level in mg/kg. Mean values without equal letters (**a-b**) differ between groups (*P* < 0.05). *FAS* Fatty acid synthetase, *INO* Source of inorganic trace minerals, *ORG* Source of organic trace minerals

**Fig. 9:**
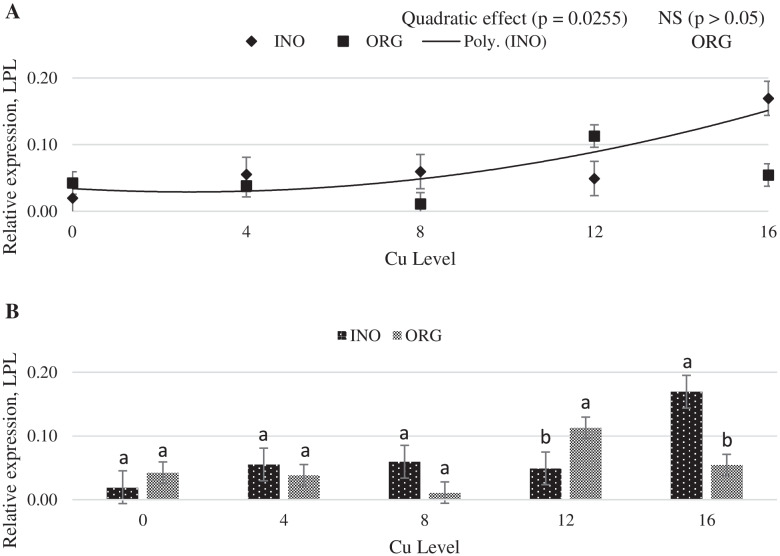
Copper levels and sources on mRNA expression of LPL gene in the broiler liver. By the unfolding of the interaction Cu levels vs sources: (**A**) Cu levels in mg/kg within the INO and ORG source, (**B**) Sources within each Cu level in mg/kg. Mean values without equal letters (**a-b**) differ between groups (*P* < 0.05). *LPL* Lipoprotein lipase, *INO* Source of inorganic trace minerals, *ORG* Source of organic trace minerals

**Fig. 10:**
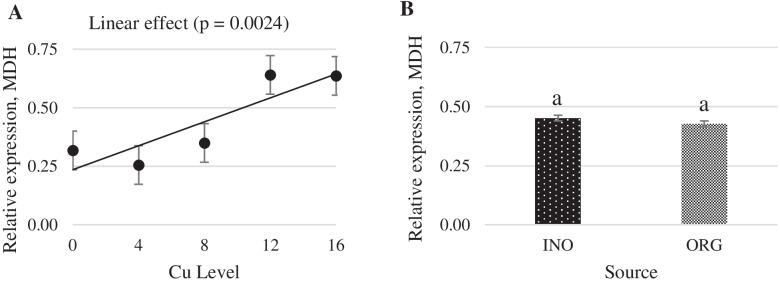
Copper levels and sources on mRNA expression of MDH gene in broiler liver. By the grouped means of the factors (Cu Level vs Source): (**A**) Cu levels in mg/kg of the grouping of sources (INO and ORG), (**B**) Sources of grouping of Cu levels. Mean values without equal letters (**a-b**) differ between groups (*P* < 0.05). *MDH* Malate dehydrogenase, *INO* Source inorganic trace minerals, *ORG* Source of organic trace minerals

Increasing dietary supplemental Cu levels elicited a linear increase (*P* < 0.05) in MT mRNA expression in chick liver, but no effect (*P* > 0.05) of trace minerals source occurred (Fig. 6). CuZnSOD mRNA expression in liver was quadratically increased as supplemental Cu levels increased (*P* < 0.05), and chicks fed CuPro and organic trace minerals exhibited a higher value (*P* < 0.05) compared with birds fed CuSO_4_.5H_2_O and inorganic trace minerals (Fig. 7). Interactive effects between supplemental Cu levels and sources (*P* < 0.05) were found for fatty acid synthase (FAS) and lipoprotein lipase (LPL) mRNA expression (Figs. 8 and 9). FAS mRNA expression was quadratically affected by level of CuPro supplementation (*P* < 0.05), and at the levels of 8 and 12 mg Cu/kg diet, chicks fed CuPro diets exhibited a lower (*P* < 0.05) FAS mRNA expression compared with chicks fed CuSO_4_.5H_2_O (Fig. 8). A quadratic effect (*P* < 0.05) on the mRNA expression of LPL in chick liver was identified in birds fed increasing levels of CuSO_4_.5H_2_O (Fig. 9). The mRNA expression of LPL was greater (*P* < 0.05) in the liver of chicks fed CuPro at 12 mg Cu/diet, but lower at 16 mg Cu/ kg diet (*P* < 0.05) compared with chicks fed CuSO_4_.5H_2_O and inorganic minerals supplemented diets (Fig. 9). Supplemental Cu levels increased (*P* < 0.05) the malate dehydrogenase (MDH) mRNA expression in chick liver, but there was no effect (*P* > 0.05) of mineral source (Fig. 10).

### MT, CTR1, STEAP-1 and ATOX 1 Relative gene expression in the small intestine

**Fig. 11:**
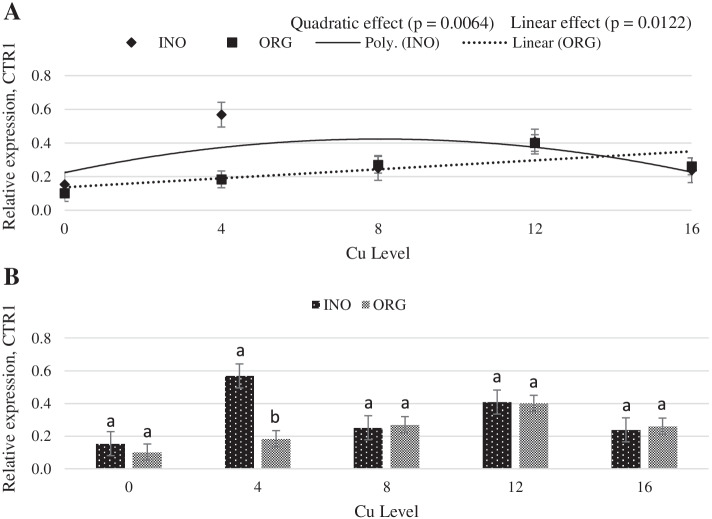
Copper levels and sources on mRNA expression of MT gene in the broiler intestine. By the unfolding of the interaction Cu level vs Source: (**A**) Cu levels in mg/kg within the INO and ORG source, (**B**) Sources within each Cu level in mg/kg. Mean values without equal letters (**a-b**) differ between groups (*P* < 0.05). *MT* Metallothionein, *INO* Source of inorganic trace minerals, *ORG* Source of organic trace minerals

**Fig. 12:**
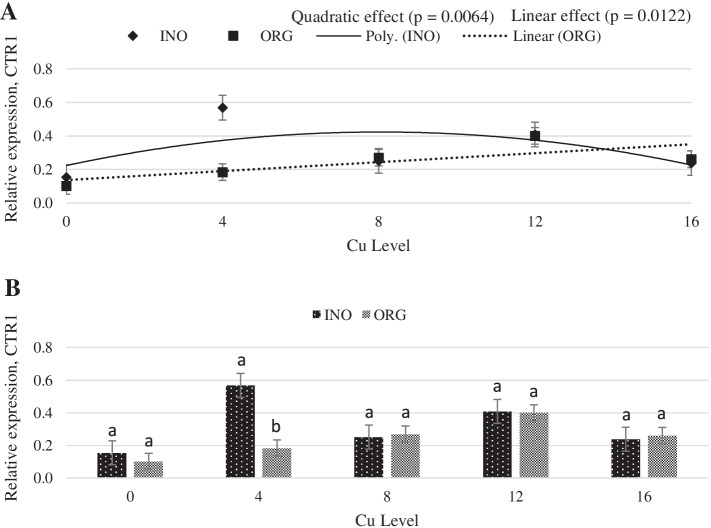
Copper levels and sources on mRNA expression of CTR1 gene in broiler intestine. By the unfolding of the interaction Cu level vs source: (**A**) Cu levels in mg/kg within the INO and ORG source, (**B**) Sources within each Cu level in mg/kg. Mean values without equal letters (**a-b**) differ between groups (*P* < 0.05). *CTR1* Copper conveyor 1, *INO* Source of inorganic trace minerals, *OR*G Source of organic trace minerals

**Fig. 13:**
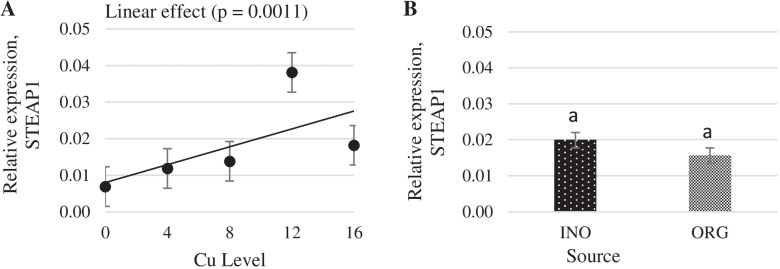
Copper levels and sources on mRNA expression of STEAP-1 gene in broiler intestine. By the pooled means of the factors (Cu Level and Source): (**A**) Cu levels in mg/kg of the grouping of sources (INO and ORG), (**B**) Sources of grouping of Cu levels. Mean values without equal letters (**a-b**) differ between groups (*P* < 0.05). *STEAP-1* Metalloreductase STEAP 1, *INO* Source of inorganic trace minerals, *ORG* Source of organic trace minerals

**Fig. 14:**
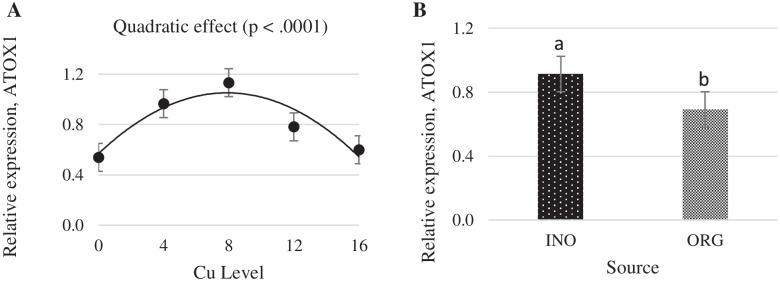
Copper levels and sources on mRNA expression of ATOX 1 gene in broiler intestine. By the pooled means of the factors (Cu Levels and Source): (**A**) Cu levels in mg/kg of the grouping of sources (INO and ORG), (**B**) Sources of grouping of Cu levels. Mean values without equal letters (**a-b**) differ between groups (*P* < 0.05). *ATOX 1* Antioxidant target 1 copper chaperone, *INO* Source of inorganic trace minerals, *ORG* Source of organic trace minerals

Interactive effects between supplemental Cu levels and trace mineral source were found (*P* < 0.05) for MT and Copper transporter 1 (CTR1) mRNA expression (Figs. 11 and 12). Chicks fed CuPro supplemented diets exhibited a linear decrease (*P* < 0.05) in MT mRNA expression in jejunum as Cu supplementation increased, whereas in CuSO_4_.5H_2_O fed chicks, jejunal MT mRNA expression changed quadratically (*P* < 0.05) with the maximum value at 12 mg Cu/kg diet. mRNA expression of MT in jejunum was greater (*P* < 0.05) in chicks fed CuPro diets at 4 mg Cu/kg diet compared with birds fed CuSO_4_.5H_2_O supplemented diets; however, at 8 and 12 mg Cu/kg diet, chicks fed CuSO_4_.5H_2_O diets exhibited a higher MT mRNA expression (*P* < 0.05) compared to the CuPro group (Fig. 11). As detailed in Fig. 12, CuSO_4_.5H_2_O and CuPro supplementation elicited a quadratic and linear increase, respectively, in the expression of the CTR1 gene in chick jejunum. At the supplemental level of 4 mg/kg diet, CTR1 gene expression was greater (*P* < 0.05) in the jejunum of chicks fed CuSO_4_.5H_2_O compared with the CuPro group (Fig. 12). Increasing dietary Cu supplementation elicited a linear (*P* < 0.05) and quadratic increase (*P* < 0.05), respectively in transmembrane epithelial antigen of the prostate (STEAP-1) (Fig. 13) and antioxidant target 1 copper chaperone (ATOX 1) mRNA expression (Fig. 14) in chick jejunum. ATOX 1 mRNA expression was greater (*P* < 0.05) in the jejunum of chicks fed CuSO_4_.5H_2_O and inorganic trace minerals compared with CuPro and organic trace minerals (Fig. 14).

## Discussion

We designed the current assay to investigate broiler responses to dietary supplemental Cu levels in inorganic or organic trace mineral supplements and establish the concentrations of Cu required to optimize performance responses with supplemental trace minerals as organic or inorganic sources. We hypothesized that when provided as CuPro, lower supplemental Cu levels would produce similar responses to higher amounts of Cu provided as CuSO_4_.5H_2_O. Additionally, based on genetic improvements, it was theorized that the requirements of dietary Cu to support optimum growth in broilers would be higher than the 8 mg/kg described by NRC [11].

To illustrate the extent to which growth, tissue mineralization and physiological responses are affected by dietary Cu and estimate the optimum dietary supplemental Cu for such responses with regression analysis, Cu supplemental levels ranged from 0 to 2 times the level recommended by NRC [11], i.e., 16 mg/kg diet. Overall, as expected, performance measures were improved as dietary Cu concentration increased in experimental diets, which confirms the essentiality of Cu for broiler metabolism. Conversely, Hu et al. [36] did not observe any effects of Cu supplementation (0 to 15 mg/kg diet) on the performance responses of broilers fed a corn-soybean meal diets supplemented with copper sulphate. In the current research, neither BW nor ADG was affected by the Cu sources investigated. Such outcomes support previous research findings that CuSO_4_.5H_2_O and organic Cu do not differ with regards to growth rate in broilers [32] and White Pekin ducks [37]. Although Cu sources did not influence the growth rate in our study, ADFI and FCR were affected by the trace mineral sources and Cu supplemental levels. Chicks fed diets supplemented with CuPro and organic trace minerals exhibited improvements of approximately 3% in FCR (1.27 vs. 1.31) and tended to have a lower feed intake compared with those fed inorganic minerals. These outcomes support previous results reported by Wen et al. [32], who found that Cu-methionine supplementation at 40 mg Cu/kg diet improved broiler FCR slightly compared with diets without supplemental Cu (8 mg Cu/kg diet) or diets containing 40 mg Cu/kg provided as copper sulfate. Similarly, Das et al. [38] reported that broiler chicks fed diets supplemented with Cu-proteinate exhibited better FCR than birds fed diets in which copper sulfate was the Cu source. In the current study, as supplemental Cu concentration increased, ADFI was linearly decreased and FCR improved up to 8–12 mg Cu/kg diet. Previously, Das et al. [38] found a linear improvement in broiler FCR as Cu dietary concentration increased. Contrary to our hypothesis, Cu source did not influence Cu concentration in broiler chick liver, breast muscle or tibia. Our findings differ from those described results reported by Attia [37] that White Pekin ducks fed organic Cu exhibited higher liver Cu concentration.

Similarly, Wu et al. [34] did not notice differences in Cu concentration in the breast muscle and liver of broilers fed diets with at 0, 10 and 20 mg/kg as copper sulphate, tribasic copper chloride or Cu-methionine. Nonetheless, as expected, we noticed a linear increase in chick liver Cu concentration as Cu supplementation increased. In the current study, the highest supplemental Cu level investigated increased 14% Cu concentration in liver compared with control. These outcomes support previous findings that Cu supplementation influences liver Cu storage in broilers [32, 34, 36, 39–41]. The liver is the primary Cu storage organ, being responsible for regulating this mineral's status in the body [42]. When Cu intake exceeds metabolic needs and liver storage reaches a critical point at which hepatic injury may occur, Cu is released into the bloodstream to be accumulated in other organs such as muscles and bones [40], as our outcomes demonstrated in chick tibias where Cu content tended to increase linearly as Cu supplementation increased (Table 4). Similarly, Adegbenjo et al. [43] and Hamdi et al. [40] reported that dietary Cu supplementation elicited an increase in the storage of Cu in the tibia of cockerels and broilers, respectively. Copper is required for bone metabolism as a co-factor for lysyl oxidase [44], which is responsible for initiating the cross-linkage formation in elastin and collagen in bones and other connective tissues [45].

As dietary Cu supplementation increased, retention of P in chick tibia tended to increase. A positive correlation between levels of Cu supplementation and the retention of P and Ca were observed by Muszyński et al. [10] in growing broiler bones and by Attia et al. [46] with breeder hens using higher Cu levels. Presumably, our responses might be associated with the solubility of the sources under study. Contrary to organic trace mineral sources, inorganic salts, such as copper sulfate, have a higher solubility in pH values similar to those observed in small intestine [47, 48], which could increase mineral-phytate complexes resistant to the hydrolytic activity of phytases. Persson et al. [49] reported that excessive copper sulfate decreases the hydrolysis of resistant phytate complexes. Banks et al. [50] noticed that when provided as Cu citrate or Cu sulfate, Cu supplementation decreased apparent P retention in broilers compared with organic Cu-lysine, which suggests a greater efficiency of organic trace minerals. Even though, we formulated a semi-purified diet to produce diets marginally deficient in Cu, both sodium phytate and exogenous phytase were added to basal diets to simulate cereal-based-diets used under commercial conditions. Therefore, aforementioned scientific evidence published about interactions between Cu sources and microbial phytase activity could be considered to explain our outcomes.

In the current research, we noticed that by adding organic Cu and trace minerals in chick feeds, Zn and Mn content in bird tibia were increased by approximately 4.5 and 6%, respectively. Both of these trace elements are indispensable for proper bone and cartilage development. Whereas Zn is involved in collagen synthesis, one of the major structural proteins of cartilage and bones [51], Mn is a co-factor for glycosaminotransferases, which attach glycosaminoglycans to a protein core to form proteoglycans [52, 53], such as chondroitin sulfate, responsible to cartilage compression resistance [54]. Chicks fed organic Cu consumed, on average, only 1.36% less than birds fed inorganic source, which indicates that the amounts of Cu consumed was similar between organic and inorganic Cu supplemented birds. Based on such findings, the better retention of Zn and Mn in tibia in chicks fed organic minerals confirm the hypothesis of lower agonistic interactions in the gastrointestinal tract, when minerals are provided in organic form compared with inorganic. Our outcomes demonstrated that organic Cu supplementation and organic trace minerals resulted in approximately 20% less Fe retained in liver. Our findings can be supported by Wu et al. [34] who observed lower Fe hepatic retention with Cu supplementation as Cu-methionine and tribasic Cu chloride and intermediate values with Cu sulphate compared with control non supplemented feed. Hepatic Fe metabolism is regulated through ceruloplasmin (CP), a Cu-dependent enzyme responsible for catalyzing the oxidation of Fe2 + to Fe3 + , which allows Fe to bind to Apo-Transferrin to be delivered from the liver to all tissues/cells of the body [55]. Welch et al. [56] have shown that Cu deficiency led rodents to accumulate Fe in liver, which is presumably related to impaired CP activity. Jarosz et al. [27] demonstrated that Cu supplementation increased serum CP concentration in growing broilers, and that such responses were greater in cooper-glycine chelate fed birds compared with birds fed Cu sulfate diets. Presumably, CuPro increased serum CP concentration compared to copper sulfate in the present experiment and that may have altered the liver Fe concentration.

Inorganic Cu and trace minerals consistently resulted in increased the activities of the antioxidant enzymes SOD and GSH-Px than did organic minerals, which suggests that inorganic minerals have greater pro-oxidant activity compared with organic ones. Such theory may be supported by the trend we found in the MDA concentration, a biomarker of lipid peroxidation. Our findings show that the MDA was approximately 55% higher in breast muscle of chicks fed inorganic Cu compared with organic. Presumably, the greater oxidative stress with inorganic minerals triggers the production of the protective antioxidant enzymes. Koslosky et al. [57] reported an increase the blood concentration of MDA and the activity of the antioxidant enzymes catalase and GSH-Px as copper nanoparticle was gradually replaced by copper sulfate in turkey diets. In the present experiment, GSH-Px activity in breast muscle increased linearly in chicks fed inorganic minerals and did the opposite behavior in birds fed organic minerals. Curiously, a difference in GSH-Px activity in breast muscle of chick was noticed in diets without supplemental Cu, which suggests that such response was due to the sources of other trace minerals in the premix used to produce treatments. All of this supports the theory that inorganic sources may induce oxidative stress in tissues compared with organic minerals.

The Cu levels and microminerals sources affected the mRNA expression of several genes related to fat and protein metabolism. Those effects may help to understand FCR responses. The AKT-1 is involved in protein metabolism as one of major signaling pathways control protein synthesis [58], and an increase in its mRNA expression associated with PI3K/Akt signaling pathway and an increase in muscle growth potential were identified in broilers with better feed efficiency [59]. Although AKT-1 expression has been shown to be affected by dietary Cu [60], in the present study, AKT- expression in chick breast muscle was higher when copper was supplemented at 8 ppm. This result can be correlated with better feed efficiency obtained when Cu were supplemented at 9.87 and 8.84 for CuPro and CuSO_4._5H_2_O, respectively.

The mTOR is a serine/threonine protein kinase [61] which regulates protein synthesis, cell growth, and autophagy in cells by receiving stimulated signals from nutrients, growth factors, and environmental stressors [62]. The mTOR gene is an important marker of protein metabolism because it is involved in the stimulation of protein synthesis by activating p70S6K, which activates other factors involved in translation initiation and elongation [63]. The results of the present study demonstrated that there is a quadratic effect of different levels of dietary Cu (independently of the source) on the mRNA expression of mTOR in breast muscle. These results are in accordance with Yang et al. [61], who reported that Cu increased the expression of mTOR in chicken liver. Further, CuPro and organic minerals increased mTOR expression when compared to CuSO_4_.5H_2_O and inorganic minerals, which may contribute to the improved FCR with organic minerals.

The GSK-3β mRNA expression is correlated with increased mRNA expression of eIF4E and tends to increase p70S6K; these proteins are involved in protein synthesis [64]. In the present study, there was a quadratic effect of CuSO_4_.5H_2_O on the expression of GSK-3β indicating that CuSO_4_.5H_2_O affected the expression of this gene and at higher levels causes a down regulation, while CuPro maintained the GSK-3β mRNA expression in the same amount regardless of the level. This result can be correlated to the better FCR observed in broilers receiving diets with CuPro. These data suggest that dietary Cu as an organic trace mineral source can support protein synthesis in broiler breast muscle, benefiting early performance.

The MT is a low molecular weight protein (6.1 kDa) found in the cell cytosol and produced in response to high levels of Cu and Zn and heavy metals such as Cd^2 +^ and Hg^2 +^ [65]. Within the intestinal cell, most of the absorbed Cu (about 80%) is retained in the cytosol, and the majority is bound to MT [66]. The MT has high affinity for Cu, and MT mRNA expression is increased by high Cu concentration [67]. In the present study, the mRNA expression of the MT was influenced by treatment in all tissues studied (breast muscle, intestine, and liver) but in different ways, which warrants further investigation. At a dietary Cu level of 4 mg/kg, birds fed CuSO_4_.5H_2_O had higher mRNA expression of MT in breast muscle compared to CuPro, but in intestine, birds fed CuPro higher mRNA MT expression compared to CuSO_4_.5H_2_O. Previous research demonstrated that Cu increases MT [68, 69]. Fry et al. [70] reported a tendency of increase in MT mRNA expression in pigs fed two different inorganic Cu sources compared with not supplemented animals.

Cu absorption in the small intestine is saturable, and some of the molecules responsible for the kinetics have been identified in the last decade. The luminal Cu is reduced to the cuprous form (Cu ^+^) by a reductase before Cu is absorbed. Some enzymes involved in this process are cytochrome B reductase 1 and STEAP-1 reductase [71]. We noticed a linear effect of Cu level on STEAP-1, an enzyme involved in Cu absorption. Such outcomes indicate that within the levels studied, Cu supplemental levels linearly increase the copper reduction potential to be absorbed.

After reduction, Cu enters the enterocytes via CTR1 [72]. Previous studies report that copper uptake by eukaryotic cells is regulated by CTR1, which is primarily responsible for copper entry and high affinity copper ingress into mammalian cells [73]. In the present study, CuSO_4_.5H_2_O and inorganic trace minerals fed at 4 mg/kg increased CTR1 expression in the intestine compared to the same level of CuPro. As the level of CuSO_4_.5H_2_O increased to 16 mg/kg there was a reduction in CTR1 expression, and the down regulation in gene expression is in agreement with Zhang et al. [74]. Contrary with CuPro the CTR1 gene expression increase linearly, suggesting that in some extent this absorption mechanism can be used. However, other authors reported that Cu concentration and source did not affect CTR1 expression in pig small intestine [70, 75].

The metallochaperone ATOX 1 is involved in the secretory pathway of Cu and its main role is to transfer Cu to the ATP7A and ATP7B Cu transport ATPases located in the trans-Golgi network and endocytic vesicles [70]. This process allows the maturation of Cu-dependent enzymes within the secretory pathway and maintains Cu levels in the cytosol and mitochondria [76]. In the present study, there was a quadratic effect of different levels of dietary Cu on the expression of ATOX 1, indicating a limit on the increase of expression of this gene in response to increasing levels of copper consumed by broilers. These results are in agreement with Zhang et al. [74] where nano Cu treated epithelial cells expressed more ATOX 1 compared with no copper supplementation.

Our findings on expression profile of differential genes STEAP 1, ATOX1, CTR1 and MT, confirms the involvement of STEAP 1, CTR1, ATOX and MT in the mechanisms of copper absorption, transport, and storage in broilers intestinal segment. However, the significant interactions between copper supplementation levels and the sources verified on the expression of CTR1 and MT genes and the differences between the sources for the ATOX1 gene expression suggest that different mechanisms are used for the absorption, transport and storage of copper minerals in different forms and require further investigations.

The antioxidant defense system depends on substances that protect the organism against damage caused by ROS [77]. Superoxide dismutase (SOD) is an important enzyme that helps against ROS damage by catalyzing the dismutation of two superoxide radicals to hydrogen peroxide and oxygen [78]. Although Cu is an essential component of SOD [79], research findings suggest that excessive Cu exposure may increase cellular ROS production [80, 81]. In the current research, the highest dose of dietary Cu investigated was 16 mg/kg diet, which may not have been high enough to cause serious increased in oxidative stress.

In breast muscle the CuZnSOD mRNA expression as a function of Cu levels in each trace mineral source exhibited a similar shape response but were up regulated until 8 mg/kg with CuSO_4_.5H_2_O and 12 mg/kg with CuPro. In liver tissue mRNA expression level for the antioxidant enzyme was also dependent on Cu levels. Higher CuZnSOD mRNA expression was verified in broilers fed organic trace minerals in liver and at some dietary Cu levels in muscle. The organic trace minerals resulted in greater CuZnSOD expression but lower enzyme activity in both tissues studied, suggesting complexity in the regulation of activity of this enzyme. Fry et al. [70] observed an increase in CuZnSOD expression when pigs were fed dietary Cu. On the other hand, Zhang et al. [74] observed a reduction in CuZnSOD expression as pigs were fed increasing doses of nano Cu.

Many regulatory factors and pathways are involved in the utilization of fatty acids. Acetyl-CoA carboxylase, FAS and carnitine palmitoyltransferase are the main enzymes involved in regulation of fatty acid synthesis and oxidation [82]. In the present study, the expression of FAS was influenced by CuPro levels and was lower at 8 and 12 mg/kg dietary Cu than when copper sulfate was fed. Additionally, the supplemental levels of inorganic Cu did not alter FAS expression, which supports previous findings reported by Lei et al. [30], but differs from Konjufca et al. [83] who reported a positive correlation between dietary Cu supplementation and broiler liver FAS activity. Hormone-sensitive lipase and lipoprotein lipase (LPL) are enzymes involved in lipid metabolism by limiting fatty acid turnover in the adipose tissue [84]. Lipoprotein lipase is the main enzyme that mediates fatty acid uptake from circulating lipoproteins in peripheral tissues [85]. In the present study, the expression of LPL in the liver was up-regulated with higher levels of CuSO_4_.5H_2_O. Lei et al. [30] reported that in the skeletal muscle, the LPL gene expression was increased by Cu supplementation, which supports our findings for dietary inorganic Cu in higher level. Our findings indicated that supplemental Cu, regardless of the sources investigated, impacted the mRNA expression of the lipogenic enzymes FAS, LPL, MDH, more specifically, the upregulation of LPL in higher levels of inorganic Cu and a down regulation of FAS in higher levels of organic Cu. These findings are in line with published literature that nutritional and pharmacological Cu levels and different Cu sources affect serum and/or plasmatic lipid fractions in birds [28, 32–34, 46, 86]. The 0 mg/kg Cu level is important for correlating the effect of other inorganic and organic trace minerals. In this study, we observed that 0 mg/kg of Cu did not provide statistical difference in any mRNA expression of genes studied, showing that the main effects are related with different Cu sources and Cu levels. As an attempt of decreasing Cu storage in body tissues of chicks, which would increase the magnitude of responses to concentrations and sources of Cu, a basal diet marginally deficient in Cu was provided to chicks in the pre-experimental phase. At the beginning of the assay, initial body weight was approximately 22% lower than targets described by the genetic strain guideline [87]. This suggests that the marginal deficient pre-starter diet in dietary Cu concentration constrained growth and, presumably, reduced Cu reserves in chick body tissues at the first 7 days post-hatch.

To establish the ideal supplemental levels for CuPro in organic trace minerals and CuSO_4_.5H_2_O in inorganic trace minerals for performance optimization, collected data were fitted to polynomial quadratic regression models. Even though the data have not fitted as well as expected to regression models, the estimates of supplemental Cu provided as CuPro and CuSO_4_.5H_2_O, which optimized responses assessed are detailed in Table 4. Although we had hypothesized that the Cu requirements of fast-growing broiler strains would be higher than those described by NRC [11], our requirement estimates are all in a narrow range near the NRC estimate of 8 mg/kg diet. These results do not clearly distinguish between the requirement estimates for the two sources of Cu, although FCR was slightly better and feed intake slightly lower on the diets containing CuPro.

## Conclusions

CuPro and organic trace minerals showed beneficial effects on the efficiency of feed utilization and on bone mineral content of growing broilers. The present results on relative gene expression related to protein metabolism, Cu storage and transport, oxidative status, and lipid metabolism indicate that different levels and sources of Cu and trace minerals alter mRNA expression in breast muscle, intestine, and liver, suggesting the different results observed in feed utilization can be related to factors beyond absorption and transport across the membrane. Dietary Cu concentrations required for optimizing growing broilers feed efficiency were estimated in the range of 8.4 to 9.9 mg Cu/kg diet.

## Methods

### The current study was carried out in compliance with the ARRIVE guidelines

The procedures involving animal care and use were previously approved by the Ethics on Animal Use Committee of the Federal University of Viçosa, Viçosa, Minas Gerais, Brazil, prior to the beginning of the assay (Register number 111/2014).

### Animals and housing

A total of five hundred 1-d-old male Cobb 500 chickens were obtained from a local commercial hatchery and used in the current assay. From 1 to 7 d of age, birds were fed a pre-starter diet formulated to meet or exceed nutritional recommendations of Rostagno et al. [88], except for Cu, whose dietary supplementation was provided at 4 mg Cu/kg of feed as copper sulfate (CuSO_4_.5H_2_O), resulting in a total dietary Cu concentration of 9 mg/kg, slightly more than the NRC [11] requirement estimate. The pre-starter diet contained less Cu than typically used in commercial production to avoid excessive reserves of this mineral. Throughout the entire pre-experimental period, chicks had free access to water and feed (mash). At 8 d of age, chicks were housed in an environmentally controlled room and allotted into 49 cm × 27 cm × 33 cm (length x height x width) plastic cages with raised wire floors until the end of the feeding assay. Initial stocking density corresponded to 30.9 chicks/m^2^. Experimental feeds (mash) and demineralized water were provided ad libitum throughout the 9-day experimental period. Photoperiod was set at 12 h light/12 h darkness. Prior to the experimental period, all chicks were weighed and assigned to treatment groups so initial body weight (167.6 ± 1.58 g) was similar among experimental treatments. Ten replicate cages of 5 chicks were randomly assigned to each of 10 treatment groups. Each cage was considered an experimental unit.

### Experimental diets and treatments

A 2 × 5 fractional factorial arrangement was used to investigate the effect of 2 sources of Cu (organic and inorganic), each at 5 supplemental Cu levels (0, 4, 8, 12, and 16 mg Cu/kg feed). Copper sources were Cu sulfate (CuSO_4_.5H_2_O, 24.5% Cu) and Cu proteinate (Bioplex® Cu, 10% Cu—Alltech, Maringá, Brazil). A semi-purified basal diet, based on casein, albumin, corn, and dextrose (Table 7), was formulated to meet or exceed nutritional requirement estimates of Rostagno et al. [88] for starter broilers (8–21 days of age), except for trace minerals. From the basal diet, 4 different diets were produced. The 4 diets differed from each other with regard to amount and source of supplemental Cu (CuSO_4_.5H_2_O or Bioplex® Cu) and to the trace mineral supplement (organic trace minerals or inorganic trace minerals) as follows: 1) basal diet with organic trace mineral supplement (ORG) without supplemental Cu; 2) ORG + 16 mg Cu/kg feed supplemented as Bioplex® Cu; 3) basal diet supplemented with inorganic trace mineral supplement (INO) without supplemental Cu; and 4) INO + 16 mg Cu/kg of feed supplemented as CuSO_4_.5H_2_O. Diets supplemented with organic trace mineral supplement containing 0 and 16 mg Cu/kg as Bioplex® Cu were mixed to produce five dilutions, resulting in five different supplemental organic Cu concentrations: 0, 4, 8, 12, and 16 mg Cu/kg of feed. The same procedure was adopted with diets supplemented with inorganic trace mineral supplement, resulting in the same levels of supplemental inorganic Cu. Organic trace microminerals (ORG) were supplied as Bioplex®Fe (15% Fe), Bioplex®Zn (15,05% Zn), Bioplex®Mn (13,86% Mn), and selenium yeast as Selplex® (0,236% Se), whereas inorganic trace minerals (INO) sources included iron sulfate (21,91% Fe), zinc sulfate (22,95% Zn), Manganese sulfate (30,9% Mn), and sodium selenite (51,6% Se), and I was added as calcium iodide (86% I) in both trace mineral supplements. Except for Cu, all trace minerals were supplied to meet or exceed NRC [11] recommendations. Sodium phytate was added to the semi-purified diet to simulate practical cereal-based-diets. All test diets were supplemented with a commercial microbial phytase enzyme to simulate typical practice. Prior to the beginning of the assay, diets were analyzed for Cu concentration (Table 1) as described by AOAC [86] (method 968.08). Overall, the measured levels of Cu were near the expected concentrations.

**Table 7:** Ingredient composition of the semi-purified basal diet (as fed basis)

Ingredients	g/kg
Corn	300
Dextrose	133
Starch	130
Albumin^a^	120
Broken rice	80.0
Cellulose^a^	40.0
Casein^a^	40.0
Soybean protein isolate	40.0
Soybean oil	20.0
Calcium carbonate^a^	16.95
Potassium phosphate^a^	14.85
Magnesium chloride^a^	6.50
Potassium chloride^a^	4.68
Choline chloride (f0%)	3.75
Mixture of amino acids^b^	35.55
Micronutrients^c^	12.20
Microminerals^d^	2.000
Phytase^e^	0.100
* Calculated nutrients*	
AMEn, kcal/kg	3128
Crude protein^f^, %	22.58
*SID amino acids, %*	
Lysine, %	1.25
Methionine, %	0.55
Methionine + Cystine, %	0.91
Threonine, %	0.83
Calcium^f^, %	0.85
Total P^f^, %	0.56
Available P, %	0.40

^a^P.A purism reagent exceeds standard ACS specification in trace metals analysis

^b^0.03% L-lysine (79%); 0.27% L-arginine (98.5%); 0.40% L-glycine (98.5%); 0.85% L-alanine (99%); and 2.0% L-glutamic acid (100%). The amino acids alanine, glycine, and glutamic acid were added to maintain the ratio of essential nitrogen to total nitrogen at 0.50

^c^0.055% coccidiostat; 0.010% avilamycin; 0.030% BHT; 1.02% sodium phytate; and 0.150% vitamin blend supplemented per kg of feed: vitamin A, 9750 IU; vitamin D_3_, 2470 IU; vitamin E, 36.6 IU; vitamin B_1_, 2.6 mg; vitamin B_2_, 6.5 mg; vitamin B_6_, 3.64 mg; vitamin B_12_, 15 mcg; vitamin K, 1.95 mg; nicotinic acid, 39.0 mg; pantothenic acid, 13.0 g; folic acid, 0.91 mg; and biotin, 0.091 mg

^d^Mineral blend supplemented per kg of feed: 80 mg Fe; 10 mg Cu; 40 mg Zn; 0.3 mg Se and 1 mg I. Except for Cu added according each experimental treatment as 0; 4; 8; 12; and 16 mg Cu. Inorganic micromineral sources: Ferrous Sulphate (21.91% Fe); Zinc Sulphate (22.95% Zn); Copper Sulphate (25.13% Cu); Sodium Selenite (51.6% Se); Calcium Iodide (86% I) and Manganase Sulphate (30.3% Mn) and Organic micromineral sources: Bioplex®Fe (15% Fe); Bioplex®Zn (15.05% Zn); Bioplex®Cu (11.36% Cu); Selenium yeast (0.236% Se); Calcium Iodide (86% I); and Bioplex® Mn (13.86% Mn)

^e^Microbial phytase, 600 FTU/kg

^f^Analyzed value

### Performance and sample collection for tissue mineralization, enzyme activity, and lipid peroxidation analysis

At 17 d of age, all chicks and feed leftovers from each experimental unit were weighed to determine body weight (BW) and average daily feed intake (ADFI), from which average daily gain (ADG) and feed conversion ratio (FCR) were calculated. Mortality rate was also monitored, to adjust FCR. At the end of the assay, one bird per cage (10 birds/treatment) was randomly selected and sacrificed by cervical dislocation without previous anesthesia. A longitudinal incision was made in the abdominal cavity to collect liver, and the left and right breast muscles. The left tibia was also collected. The liver and muscle tissues were lyophilized for 72 h at − 80ºC under 800 mbar of pressure (Liobras– São Carlos, SP), ground in a ball mill (Tecnal Equipamentos para Laboratório, TE-350, São Paulo, Brazil), and stored for further analysis of mineral content. After appropriate dilution, Cu, Zn, Mn, Fe, Ca, and P contents were estimated by atomic absorption spectrophotometer (Spctr AA-800; Varian spectrometer, Harbor City, CA) in the Animal Nutrition Laboratory (Federal University of Viçosa, Viçosa, MG, Brazil) using AOAC official method 968.08 [86]. Tibias were ether extracted for 4 h in a Soxhlet extractor (method 920.29) as described by AOAC [86], ground, and analyzed for mineral concentration by the same procedures as breast muscle and liver samples.

The superoxide dismutase (SOD) and glutathione peroxidase (GSH-Px) activities in tissues were measured according to Walsh et al. [89] using the kits of Randox Laboratories Ltda. (County Antrim, UK) following the manufacturer’s guidelines. The extent of lipid peroxidation in breast muscle was assessed as thiobarbituric acid reactive substances (TBARS) and reported as concentration of malondialdehyde (MDA), which was used as standard according to the method described by Beuge and Aust [90].

### Gene expression: sample collection, RNA extraction and Real-Time Quantitative PCR

At 17 d of age, 1 chick from each of 4 replicates (4 chicks/treatment) was randomly selected and sacrificed by cervical dislocation without previous anesthesia. Breast muscle, liver, and intestine (jejunum-ileal junction) samples were collected immediately, ground, stored in individual tubes containing RNALater (Ambion, Austin, TX, USA) and frozen at -80° C for further analysis of gene expression. Total RNA from snap-frozen tissue samples of breast muscle, liver, and intestine was extracted in Trizol reagent (Invitrogen, Karlsruhe, Germany) according to the manufacturer’s protocol. Genomic DNA was removed from extracted RNA with deoxyribonuclease (Invitrogen, Karlsruhe, Germany). The purity (260:280 absorbance), concentration, and integrity of the total RNA from each sample were quantified using a Nanovue (GE electronics) spectrophotometer and gel electrophoresis (1% agarose gel and stained with ethidium bromide). Standard cDNA synthesis was achieved by GoScript Reverse Transcription (RT) system (Promega, Corp., Madison, WI) according to the manufacturer’s protocol with annealing temperature of 25 °C for 5 min and extension of 42 °C for 1 h. Three cDNA concentrations (15, 45, and 135 ng/μl) and three primer concentrations (200, 400, 800 mM) were used. The efficiency of PCR amplification was calculated for each gene, targets and housekeeping, using the following formula: E = (10 (-1/slope) -1) × 100) [35]. After efficiency analysis, the concentration of the most appropriate primers was used in the PCR reactions. Analysis of relative mRNA levels of AKT-1, mTOR, GSK-3β, MT, and CuZnSOD in the breast muscle, MT, CTR1, ATOX 1, and STEAP-1 in intestine, and MT, CuZnSOD, FAS, LPL, MDH in the liver was performed by real-time reverse-transcription PCR. The housekeeping genes, β-actin and GAPDH, were used as normalizing controls for all reactions. The annealing temperature of 60 °C was determined to be ideal for all primers. The RT-qPCR was performed by gene-specific primers (Table 8) designed using Primer Quest Tool (www.idtdna.com/Primerquest/Home/Index) available on the IDT (http://www.idtdna.com) database. The reactions were carried out according to the manufacturer’s protocol using the SYBR Green detection kit with GoTaq qPCR Master Mix (Promega, Madison, WI, USA). The RT-qPCR reaction conditions were adjusted with initial denaturation temperature at 95 °C for 2 min and 40 cycles of annealing at 60 °C (for all genes) and denaturation at 95 °C for 15 s. At the end of the amplification cycling reaction, an additional step was added with a gradual increase in temperature from 60 to 95 °C for analysis of the dissociation curve. The amplification of genes was performed in duplicate using the ABI 7300 system (Applied Biosystems, Foster City, CA, USA). Amplification efficiency ranged from 91 to 109% except for MT, in the intestine (80%), and CuZnSOD in the breast muscle (115%). The values of the coefficient of determination (R^2^) were equal to or greater than 0.98 for all genes (Table 9). The specificity of the primers was assessed by the dissociation curve, which showed only one peak, indicating that no dimer was detected and exhibited excellent performance. The results were obtained through the Sequence detection System (SDS; V.2.0.6) program (Applied Biosystems, Foster City, CA, USA) that generated the cycle threshold parameter (Ct). The Ct values were exported to Microsoft Excel to calculate the Ct mean, standard deviation, and standard curve for each gene. The relative quantification of gene expression was analyzed using the 2-ΔCt method reported by Livak and Schmittgen [91]. All real-time quantitative PCR analyses were run in duplicate.

**Table 8:** *G. gallus* genes, housekeeping genes for chickens and their specific primers used in RT-qPCR analysis

Genes	Description	Sequence 5 ' → 3'	Function
FAS	Fatty Acid Synthetase	Fw: AGCGTGCTATGCTTGCC Rv: GTCCGTGACGAATTGCTTTAT	Lipid metabolism
LPL	Lipoprotein lipase	Fw: TACAGTCTGGGTGCTCAT Rv: GCATACTCAAAGGTGGG	Lipid metabolism
MDH	Malate dehydrogenase	Fw: AAAGACTGTGAAGGTGGTAG Rv: ATCCAAGCGAGTCAAGC	Lipid metabolism
CuZnSOD	Superoxide dismutase copper-zinc	Fw: GGAGGAGTGGCAGAAGT Rv: TAAACGAGGTCCAGCAT	Antioxidant defense system
MT	Metallothionein	Fw: AAGGGCTGTGTCTGCAAGGA Rv: CTTCATCGGTATGGAAGGTACAAA	Storage and transportation
mTOR	Mechanistic target of rapamycin	Fw: GGCAATCAGTGTGCCTACCT Rv: GGCAGGCTCTTAAGGCAGAA	Protein Metabolism
GSK 3B	Glycogen synthase kinase	Fw: GGGCTGTGTATTGGCTGAAC Rv: GTAGGCGTTCCCAGAACCTT	Protein Metabolism
AKT 1	Protein kinase serine-threonine 1	Fw: ACGCTGACAGAAAACCGTGT Rv: ACAACTCCCCTCCGTTAGCA	Protein Metabolism
SLC31A1 (CTR1)	Copper transporter 1	Fw: CATCTTCAGGAGGTGGTCAT Rv: CAACGGCACATTCTCATAGC	Copper transporter
ATOX1	Antioxidant target 1 copper chaperone	Fw: AGATGTCGGAAAGGAGAGG Rv: AGAAGCAGCACCTCGAT	Copper Intracellular transporter
STEAP1	Transmembrane Epithelial Antigene of the Prostate 1	Fw: CCTATTGTGGTCCTGCTCT Rev: AGCTTCCCAACCACATCT	Reduce Cu^2+^ to Cu^1+^
β-actin	Β-actin protein	Fw: GAGAAATTGTGCGTGACATCA Rv: CCTGAACCTCTCATTGCCA	Housekeeping gene
GAPDH	Glyceraldehyde-3-phosphate dehydrogenase	Fw: CAAGTGGACATTGTCGCCATCA Rv: GCTTCCCATTCTCAGCCTGACT	Housekeeping gene

Abbreviations: *Fw* Forward, *Rv* Reverse (5′ → 3′)

**Table 9:** Parameters of gene-specific primers and reference genes obtained from the efficiency curve analysis in RT-qPCR

Tissue	Gene	T ^o^C	[cDNA]	[Primer]	Efficiency, %	R^2^	Slope
Breast Muscle	mTOR	60	45 ng/μl	200 mM	105	0.99	-3.201
	GSK 3	60	45 ng/μl	200 mM	108	0.98	-3.135
	AKT 1	60	45 ng/μl	400 mM	109	0.99	-3.131
	CuZnSOD	60	45 ng/μl	200 mM	115	0.98	-3.013
	MT	60	45 ng/μl	800 mM	109	0.99	-3.129
	β-actin	60	45 ng/μl	200 mM	91.0	0.99	-3.548
Liver	FAS	60	45 ng/μl	400 mM	94.0	0.98	-3.474
	LPL	60	45 ng/μl	400 mM	108	0.99	-3.135
	MDH	60	45 ng/μl	200 mM	104	0.99	-3.228
	CuZnSOD	60	45 ng/μl	400 mM	103	0.99	-3.145
	MT	60	45 ng/μl	400 mM	106	0.98	-3.192
	β-actin	60	45 ng/μl	400 mM	109	0.98	-3.123
Intestine ^a^	CTR1	60	45 ng/μl	400 mM	96.0	0.99	-3.412
	ATOX1	60	45 ng/μl	400 mM	101	0.99	-3.287
	MT	60	45 ng/μl	400 mM	80.0	0.99	-3.917
	STEAP1	60	45 ng/μl	400 mM	105	0.99	-3.199
	GAPDH	60	45 ng/μl	400 mM	94.0	0.99	-3.482

Abbreviations: *mTOR* Mechanistic target of rapamycin, *GSK* 3 Glycogen synthase kinase, *AKT* 1 Protein kinase serine-threonine 1, *CuZnSOD* Superoxide dismutase copper-zinc, *MT* Metallothionein, *FAS* Fatty Acid Synthetase, *LPL* Lipoprotein lipase, *MDH* Malate dehydrogenase, *CTR1* Copper transporter 1, *ATOX1* Antioxidant target 1 copper chaperone; STEAP1, Transmembrane Epithelial Antigene of the Prostate

^a^ Junction jejunum-ileum

### Statistical analysis

The cage served as the experimental unit for growth performance, while the single chick per cage represented the cage as the experimental unit for tissue mineral contents, antioxidant enzyme activity and mRNA gene expression.

Data were analyzed as a completely randomized design under an incomplete 2-way (source x levels) factorial assay with factors of inorganic versus organic trace minerals, levels of Cu from CuPro, and levels of Cu from copper sulfate. Several of the possible combinations of these factors were excluded from the experiment so the traditional two-way factorial analysis was generalized to a fractional factorial design, which consists of a carefully chosen subset (fraction) of experimental treatments [92]. This approach is easily accomplished by using common statements from PROC MIXED of SAS® software [93]. The significance (*P* < 0.05) of source effect (only two levels) was evaluated through an F-test, whereas orthogonal contrasts of linear and quadratic responses were used to assess effects of Cu levels. Finally, the supplemental level of inorganic and organic Cu which optimized biological responses assessed were estimated by using a polynomial regression model as follows: Y = (β0 × X^2)^ + (β1 × X) + β2, where Y is the dependent variable, X is the dietary supplemental Cu level, β0 is the quadratic coefficient, β1 is the linear coefficient, and β2 is the intercept. Statistical significance was considered as 0.05 and the term “tendency” is used for situations in which the *P*-value is between 0.05 and 0.1.

### Title of data

Tables: Ingredient composition of the semi-purified basal diet (as fed basis), Analyzed concentration of copper and trace minerals in experimental diets (as fed basis); *G. gallus* genes, housekeeping genes for chickens and their specific primers used in RT-qPCR analysis, Parameters of gene-specific primers and reference genes obtained from the efficiency curve analysis in RT-qPCR, Growth performance of 17 d broilers chicks fed different copper levels and sources, Optimum supplemental copper (Cu) level for broiler chicks considering sources individually or combined, Mineral concentration (dry matter) on tissues of 17 d old broiler chicks fed different copper levels and sources, Antioxidant enzyme activity and malondialdehyde concentration in 17 d old broiler chicks fed different copper levels and sources, mRNA expression in tissue of broiler fed different copper levels and sources. Figures: Copper levels and sources on mRNA expression of AKT-1 gene in broiler breast muscle, Copper levels and sources on mRNA expression of mTOR gene in broiler breast muscle, Copper levels and sources on mRNA expression of GSK-3β gene in broiler breast muscle, Copper levels and source on mRNA expression of MT gene in broilers breast muscle, Copper levels and sources on mRNA expression of CuZnSOD gene in broiler breast muscle, Copper levels and sources on mRNA expression of MT gene in the broiler liver, Copper levels and sources on mRNA expression of CuZnSOD gene in the broiler liver, Copper levels and sources on mRNA expression of FAS gene in the broiler liver, Copper levels and sources on mRNA expression of LPL gene in the broiler liver, Copper levels and sources on mRNA expression of MDH gene in broiler liver, Copper levels and sources on mRNA expression of MT gene in the broiler intestine, Copper levels and sources on mRNA expression of CTR1 gene in broiler intestine, Copper levels and sources on mRNA expression of STEAP-1 gene in broiler intestine, Copper levels and sources on mRNA expression of ATOX 1 gene in broiler intestine.

### Description of data

The responses collected in the current research are described as means of treatments in Tables from 1 to 9, and in the Figures from 1 to 14.

## Supplementary Information

**Additional file 1.** 

## Data Availability

The dataset generated and/or analyzed during the current study is not publicly available since the data is a preliminary part of another study. The data is, however, available from the corresponding author on reasonable request.

## Abbreviations

ADFI: Average daily feed intake
ADG: Average daily gain
AKT-1: Protein kinase serine-threonine 1
ATOX 1: Antioxidant target 1 copper chaperone
BW: Body weight
CP: Ceruloplasmin
CTR1 (SLC31A1): Copper transporter 1
CuZnSOD: Superoxide dismutase copper-zinc
FAS: Fatty Acid Synthetase
FCr: Feed conversion ratio
Fw: Forward
GAPDH: Glyceraldehyde-3-phosphate dehydrogenase
GSH-Px: Glutathione peroxidase
GSK-3β: Glycogen synthase kinase 3β
L: Level
LPL: Lipoprotein lipase
MDA: Malondialdehyde
MDH: Malate dehydrogenase
MT: Metallothionein
mTOR: Mechanistic target of rapamycin
NRC: National Research Council
R2: Coefficient of determination
ROS: Reactive oxygen species
Rv: Reverse Transcription
S x L: Interaction between Source and Level
S: Source
SEM: Standard error of means
SOD: Superoxide dismutase
STEAP-1: Transmembrane Epithelial Antigene of the Prostate
TBARS: Thiobarbituric acid reactive substances
β-actin: β-Actin protein
